# PM2.5 Causes Increased Bacterial Invasion by Affecting HBD1 Expression in the Lung

**DOI:** 10.1155/2024/6622950

**Published:** 2024-01-27

**Authors:** Tianqi Zheng, Yajun Wang, Zheng Zhou, Shuyang Chen, Jinjun Jiang, Shujing Chen

**Affiliations:** ^1^Department of Pulmonary and Critical Care Medicine, Zhongshan Hospital, Fudan University, Shanghai, China; ^2^Shanghai Respiratory Research Institute, Shanghai, China

## Abstract

Our research addresses the critical environmental issue of a fine particulate matter (PM2.5), focusing on its association with the increased infection risks. We explored the influence of PM2.5 on human beta-defensin 1 (HBD1), an essential peptide in mucosal immunity found in the airway epithelium. Using C57BL/6J mice and human bronchial epithelial cells (HBE), we examined the effects of PM2.5 exposure followed by *Pseudomonas aeruginosa (P. aeruginosa)* infection on HBD1 expression at both mRNA and protein levels. The study revealed that PM2.5's toxicity to epithelial cells and animals varies with time and concentration. Notably, HBE cells exposed to PM2.5 and *P. aeruginosa* showed increased bacterial invasion and decreased HBD1 expression compared to the cells exposed to *P. aeruginosa* alone. Similarly, mice studies indicated that combined exposure to PM2.5 and *P. aeruginosa* significantly reduced survival rates and increased bacterial invasion. These harmful effects, however, were alleviated by administering exogenous HBD1. Furthermore, our findings highlight the activation of MAPK and NF-*κ*B pathways following PM2.5 exposure. Inhibiting these pathways effectively increased HBD1 expression and diminished bacterial invasion. In summary, our study establishes that PM2.5 exposure intensifies *P. aeruginosa* invasion in both HBE cells and mouse models, primarily by suppressing HBD1 expression. This effect can be counteracted with exogenous HBD1, with the downregulation mechanism involving the MAPK and NF-*κ*B pathways. Our study endeavors to elucidate the pathogenesis of lung infections associated with PM2.5 exposure, providing a novel theoretical basis for the development of prevention and treatment strategies, with substantial clinical significance.

## 1. Introduction

Fine particulate matter with an aerodynamic diameter of 2.5 *μ*m or less (PM2.5), predominantly originating from industrial and vehicle emissions, is a global concern [1–3]. Numerous epidemiological and clinical studies have established a connection between PM2.5 and a variety of diseases affecting different systems [4–9]. Specifically, PM2.5 has been linked to respiratory tract infections, including influenza, bronchitis, and exacerbations of chronic obstructive pulmonary disease (COPD) [10–14]. Extensive clinical research across various populations has shown a correlation between PM2.5 exposure and increased rates of lung infections and mortality. For instance, a 10 *µg*/m^3^ increase in PM2.5 concentration leads to a 6.32% rise in emergency room visits for pneumonia and a 4.72% increase in respiratory infections [15]. Children with cystic fibrosis are at a 24% higher risk of initial *Pseudomonas aeruginosa (P. aeruginosa)* infections with increased PM2.5 exposure [16]. Furthermore, every 2 *µg*/m^3^ increase in PM2.5 levels is linked to a 3% increase in community visits for pneumonia [17], while a 20 *µg*/m^3^ increase correlates with a 0.67% increase in hospital admissions for pneumonia [18]. Collectively, these studies highlight the significant role of PM2.5 exposure in escalating lung infection risks.

The respiratory tract, constantly exposed to a plethora of external environmental potential pathogens, has garnered significant research interest. Recent advancements have enhanced our understanding of the impact of particulate matter (PM) exposure, including PM2.5 from air pollution, on lung pathogen infections. Notably, urban PM exposure leads to increased mucus production and goblet cell proliferation, causing airway obstruction and reduced pathogen clearance through mucociliary action, potentially facilitating pathogen colonization [19, 20]. Such exposure may also deplete commensal bacteria in the airways, such as species of Prevotella, Clostridium, Flavobacterium, and Vibrio, thus potentially favoring the proliferation of pathogenic bacteria like Streptococcus spp. [21–23]. Although, alveolar macrophages are the key in pathogen defense, studies indicate that macrophages with high PM uptake show decreased pathogen elimination efficiency, increasing the risk of sustained infections [24, 25]. Chronic exposure to air pollutants triggers a Th2-mediated inflammatory response, activating toll-like receptors and generating reactive oxygen species, which perpetuates inflammation and enhances vulnerability to lung pathogens [26–31]. Some researches have also suggested that PM2.5 influences cytokine secretion in the lung epithelial cells [32, 33].

Human bronchial epithelial (HBE) cells, a component of the respiratory epithelium, secrete various antimicrobial peptides, including defensins, to combat inhaled particles and pathogens [34]. The *β*-defensin family, known for their broad-spectrum antimicrobial properties, primarily function by forming channels in the lipid bilayer, disrupting bacterial cell membranes [35–38]. Human beta-defensin 1 (HBD1), expressed in human airway epithelia, is critical in enhancing the antimicrobial efficacy of airway surface fluid and in providing mucosal defense in the lungs [39]. HBD1 combats Gram-positive bacteria by obstructing the cell wall synthesis [40–42] and inhibits bacterial invasion by forming nanonets to ensnare bacteria [43]. It also protects the epithelium from colonization by commensal bacteria and opportunistic fungi [44]. A decrease in HBD1 expression correlates with an increased infection risk.

The impact of PM2.5 exposure on the downregulation of HBD1 and its regulatory mechanisms remain an underexplored area of research. Building on the previous studies, we hypothesized that PM2.5 hinders bacterial clearance by modulating HBD1 expression in the respiratory tract. Therefore, our research primarily investigates the impact of PM2.5 exposure on *P. aeruginosa* infection and HBD1 expression, delving into the associated mechanisms. This study aims to elucidate the pathogenesis of lung infections arising from PM2.5 exposure and to propose an innovative theoretical framework for the prevention and management of these conditions. The insights gained from this research have substantial clinical relevance.

## 2. Material and Methods

### 2.1. Ethics Declarations

The animal experiments were conducted in accordance with the Laboratory Animals-Guideline of welfare and ethics and were consistent with the ARRIVE guidelines. All methods used were approved by the Experimental Animal Ethics Committee of Fudan University and the Ethics Committee of Zhongshan Hospital, Fudan University.

### 2.2. *P. aeruginosa* PAO1 Preparation

*P. aeruginosa* PAO1 (PAO1) marked by GFP was given by Prof. Yuanlin Song's group at Zhongshan Hospital, Fudan University. PAO1 was cultured in *P. aeruginosa* selective medium (Chromagar, France) and was diluted with PBS for further experiment.

### 2.3. Cell Culture

The HBE cells used in this study were provided by Professor Yuanlin Song's group at Zhongshan Hospital, Fudan University. The HBE cells were cultured in RPMI 1640 medium (Cat. No. R1383, Sigma-Aldrich, USA) supplemented with FBS (Biological Industries, Israel) and penicillin–streptomycin (Cat. No. C0222, Beyotime, China). The cells were maintained in a 5% CO_2_ incubator at 37°C. Standard PM2.5 (Cat. No. SRM2786, National Institute of Standards and Technology, NIST, USA) was used as the experimental material. A solution of this standard, diluted in PBS, was added to the cells at 50%–60% confluency for 6 days prior to infection with PAO1. Cells were harvested 2 hr post-PAO1 addition. We developed PAO1 infected HBE cell models at various multiplicities of infection (MOIs of 1, 10, and 20), and exposed them to gradient concentrations of PM2.5 (0, 50, 100, and 200 *μ*g/mL). Techniques such as colony counting, laser confocal microscopy, and flow cytometry were employed to assess viable bacterial count within the cells and to examine changes in the bacterial invasion. Further details are provided below.

**Figure 1:**
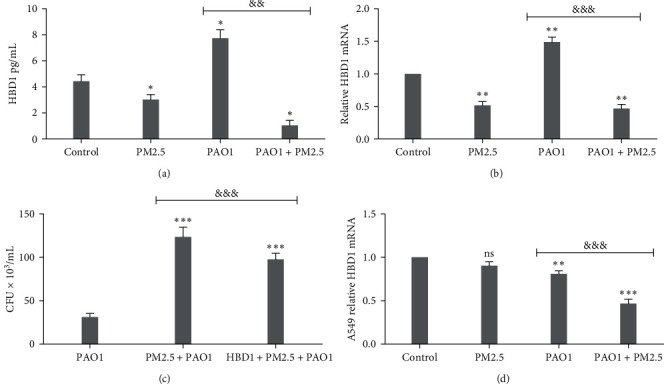
Expression of HBD1 at different conditions within the cell model. (a) ELISA was used to detect the levels of secretory HBD1 in the supernatants of HBE cell cultures in the control, PM2.5, PAO1, and PM2.5 + PAO1 groups (*n* = 3). (b) RT–qPCR was performed to detect the mRNA levels of HBE in the control, PM2.5, PAO1, and PM2.5 + PAO1 groups of HBE cells (*n* = 3). (c) Cell lysate smear plate colony count (*n* = 3). (d) RT–qPCR was performed to detect the mRNA levels of A549 in the control, PM2.5, PAO1, and PM2.5 + PAO1 groups of A549 cells (*n* = 3). (a, b, d)  ^*∗*^*P*  < 0.05,  ^*∗∗*^*P*  < 0.01,  ^*∗∗∗*^*P*  < 0.001  vs. control, ^&&&^*P*  < 0.001 PAO1 vs. PAO1 + PM2.5 (one-way ANOVA). (c)  ^*∗∗∗*^*P*  < 0.001 vs. PAO1, ^&&&^*P*  < 0.001 PAO1 + PM2.5 vs. HBD1 + PAO1 + PM2.5 (one-way ANOVA).

To assess the alterations in HBD1 expression and PAO1 invasion, this research established four cell models (HBE and A549): control, PM2.5: only exposure (100 *μ*g/mL), *PAO1*: only infection (MOI10), and combined PAO1 infection (MOI10) with PM2.5 exposure (100 *μ*g/mL). All cell models were based on HBE cells, except for those illustrated in Figure 1(d).

For the PM2.5-exposed HBE cell model, western blot analysis was conducted to assess the expression of pathway proteins. The cell models were divided into four groups: control, PM2.5 exposure (100 *μ*g/mL), PAO1 (MOI10) infection, and PAO1 (MOI10) infection plus PM2.5 (100 *μ*g/mL) exposure. Total proteins were collected and subjected to western blot experiments to detect the expression levels of MAPK/NF-*κ*B pathway proteins.

To explore the effect of MAPK/NF-*κ*B pathway inhibitors on bacterial invasion in the PM2.5 + PAO1 cell model, the experimental conditions and grouping of cells are as follows: PAO1 (MOI10) infection + PM2.5 (100 *μ*g/mL) exposure, U0126 (10 *μ*M) + PAO1 (MOI10) infection + PM2.5 (100 *μ*g/mL) exposure, SB203580 (10 *μ*M) + PAO1 (MOI10) infection + PM2.5 (100 *μ*g/mL) exposure, SP600125 (10 *μ*M) + PAO1 (MOI10) infection + PM2.5 (100 *μ*g/mL) exposure, and BAY11-7082 (5 *μ*M) + PAO1 (MOI10) infection + PM2.5 (100 *μ*g/mL) exposure. Bacterial invasion was measured in these groups.

To explore the impact of MAPK/NF-*κ*B pathway inhibitors on HBD1 expression in the PM2.5 + PAO1 cell model, the experimental conditions and grouping of cells are as follows: PAO1 (MOI10) infection + PM2.5 (100 *μ*g/mL) exposure, U0126 (10 *μ*M) + PAO1 (MOI10) infection + PM2.5 (100 *μ*g/mL) exposure, SB203580 (10 *μ*M) + PAO1 (MOI10) infection + PM2.5 (100 *μ*g/mL) exposure, SP600125 (10 *μ*M) + PAO1 (MOI10) infection + PM2.5 (100 *μ*g/mL) exposure, and BAY11-7082 (5 *μ*M) + PAO1 (MOI10) infection + PM2.5 (100 *μ*g/mL) exposure. The expression levels of HBD1 both intracellularly and extracellularly were assessed.

### 2.4. Cell Viability Assay

Cell viability was assessed using the Cell Counting Kit-8 (CCK-8) method, as per the instructions of the CCK-8 kit (Cat. No. 40203ES60, Yeason Biotech Co., China). Cells treated with PM2.5 and PAO1 were incubated with the CCK-8 solution at 37°C for 2 hr. Viability was determined by measuring the optical density (OD) at 450 nm using a microplate reader. In addition, after coculturing HBE cells with PM2.5 at concentrations ranging from 0 to 200 *μ*g/mL for periods of 0–48 hr, cell morphology and growth rate were monitored under a microscope, and viability was evaluated using the CCK-8 assay.

### 2.5. Animal Model

Male C57BL/6J mice (8−10-week old) were procured from Shanghai Jihui Company, Shanghai, China, and housed in the Experimental Animal Center at Fudan University. The study protocols were approved by both the Experimental Animal Ethics Committee and the Ethics Committee of Zhongshan Hospital at Fudan University. The PM2.5 animal models were categorized into four groups: a control group (25 *μ*L PBS daily), a PM2.5 exposure group (100 *μ*g/25 *μ*L PM2.5 daily), a PAO1 + PM2.5 group (5 × 10^6^ CFU PAO1 and 100 *μ*g/25 *μ*L PM2.5 daily), and a PAO1 + PM2.5 + HBD1 group (5 × 10^6^ CFU PAO1, 100 *μ*g/25 *μ*L PM2.5, and 1 *μ*g/25 *μ*L HBD1 daily). Mice received intratracheal instillation of the PM2.5 solution for 6 days or were infected with PAO1 via intratracheal instillation on the sixth day, as per the assigned group. Bronchoalveolar lavage fluid (BALF) and lung tissues were collected post-euthanasia. Survival was monitored at 2 hr intervals for the initial 50 hr and subsequently every 3−4 hr for the remaining 50 hr to calculate survival rates. Euthanasia was performed via an overdose of intraperitoneal avertin, ensuring deep anesthesia followed by exsanguination to confirm euthanasia.

To assess the impact of MAPK/NF-*κ*B pathway inhibitors on bacterial invasion and HBD1 expression in the PM2.5 + PAO1 animal model, the study was divided into several groups: PAO1(5 × 10^6^ CFU) infection + PM2.5 (100 *μ*g/25 *μ*l/day) exposure, U0126 (10 mg/kg) + PAO1(5 × 10^6^ CFU) infection and PM2.5 exposure, SB203580 (10 mg/kg) + PAO1 (5 × 10^6^ CFU) infection and PM2.5 exposure, SP600125 (10 mg/kg) + PAO1 (5 × 10^6^ CFU) infection and PM2.5 exposure, and BAY11-7082 (10 mg/kg) + PAO1 (5 × 10^6^ CFU) infection and PM2.5 exposure. The study measured bacterial invasion in the lungs of mice and the expression levels of HBD1.

### 2.6. Hematoxylin and Eosin (H&E) and Immunohistochemical Staining of Lung Tissues

The left lung lobe of C57BL/6J mice was excised, fixed in 4% paraformaldehyde, dehydrated, embedded in paraffin, and sectioned into 4 *μ*m slices. These sections were then stained with H&E using the HE reagent (Cat. No. C0105S, Beyotime, China). For immunohistochemistry (IHC) staining, sections were incubated with specific antibodies and developed with DAB (Cat. No. P0202, Beyotime, China). Microscopic evaluation of the sections was conducted, with scoring based on the immuno-reactive score (IRS) criteria. Two experienced pathologists independently performed blinded scoring on three sections per sample, and the average of these scores was recorded as the final lung tissue damage score and IRS.

### 2.7. Enzyme-Linked Immunosorbent Assay (ELISA)

BALF was taken and the experiment was performed according to the ELISA kit protocol (Cat. No. RX101515H, Ruixin Biotech Co., China), and the OD450 value was measured with a microplate reader.

### 2.8. Plate Count

Cells were dissociated using Trypsin-EDTA (Cat. No. 25200056, Gibco, USA) at 37°C for 15 min. The cell lysate was subjected to gradient dilution, followed by inoculation on *P. aeruginosa*-selective culture plates. Concurrently, the left lung lobe was excised from C57BL/6J mice, sectioned, and homogenized before being transferred into Eppendorf tubes. The samples were centrifuged at low speed for 3 min, and the supernatant was similarly diluted and inoculated onto culture plates. These plates were then incubated at 37°C overnight, after which the bacterial colonies were enumerated.

### 2.9. Confocal Microscopy

Treated cells were fixed in 4% paraformaldehyde and subsequently incubated with DiL (Cat. No. C1036, Beyotime, China) in the dark. Following this, cells were stained with DAPI (Cat. No. C1002, Beyotime, China) and examined under a confocal microscope (Leica, Germany).

### 2.10. Reverse Transcription–Quantitative Polymerase Chain Reaction (RT–qPCR)

RNA was isolated using TRIzol reagent (Cat. No. 15596026, Thermo Fisher Scientific Inc, USA). The extracted RNA was then utilized for cDNA synthesis employing the PrimeScript™ RT reagent Kit (Cat. No. RR037A, Takara Bio, Japan). RT–qPCR was conducted with SYBR® Green (Roche Group, Switzerland) on a Step One Plus Real-Time PCR System. Gene expression levels were quantified and analyzed based on the 2^−*ΔΔ*CT^ method. The RT–qPCR primers used are as follows: mice GAPDH forward, AGGTCGGTGTGAACGGATTTG, mice GAPDH reverse, GGGGTCGTTGATGGCAACA, mice *DEFB1* forward, AGGTGTTGGCATTCTCACAAG, mice *DEFB1* reverse, GCTTATCTGGTTTACAGGTTCCC, human GAPDH forward, CGGATTTGGTCGTATTGGG, human GAPDH reverse, CTCGCTCCTGGAAGATGG, human *DEFB1* forward, ATGAGAACTTCCTACCTTCTGCT, and human *DEFB1* reverse, TCTGTAACAGGTGCCTTGAATTT.

### 2.11. Western Blot

Tissues and cells were lysed using RIPA buffer (Cat. No. P0013B, Beyotime, China) supplemented with phosphatase and protease inhibitors (Cat. No. P1005, Beyotime, China). Proteins were quantified using a BCA kit (Cat. No. P0010, Beyotime, China) and then separated by SDS-PAGE (Cat. No. PG113, Epizyme Biomedical Technology Co., China) before being transferred to PVDF membranes (Millipore, USA). These membranes were incubated with primary antibodies against i*κ*B (39 kDa), P65 (65 kDa), P44 (42–44 kDa), P38 (40 kDa), JNK (46–55 kDa) (Cat. No. 9926 T, 8242 T, 4814 T, Cell Signaling Technology, Inc., USA), *β*-actin (45 kDa), and Lamin-B (67 kDa) (Cat. No. sc-374015, sc-47778, Santa Cruz Biotechnology, Inc., USA), diluted in a 3% bovine serum albumin (BSA) solution at a 1 : 1,000 ratio. Subsequently, they were incubated with the corresponding secondary antibodies. Protein bands were visualized using Super ECL Plus detection reagents (Cat. No. H31500, Tianjin Tiandi Renhe Biotech Co., China) and recorded using a chemiluminescence imaging analysis system (Tanon 5200, YuanPingHao Biotech, China).

### 2.12. Drug Usage

Solution preparation is as follows: PM2.5 was prepared at a concentration of 50 mg/mL using sterile PBS, vortexed thoroughly, and stored in a tinfoil-sealed refrigerator at 4°C, shielded from light. HBD1 (Cat. No. NBP2-34906-5 *μ*g, Novus Biologicals, LLC, USA) was dissolved in sterile water containing 0.02% acetic acid and 0.4% BSA to create a 1 mg/mL storage solution, and stored at −20°C. Pathway inhibitors were prepared as a 0.1 *μ*M stock solution in DMSO and stored at −20°C in a tinfoil-sealed container.

For the animal groups, the control group received 25 *μ*L of PBS solution daily via airway administration. Mice in the PM2.5 group were administered 25 *μ*L of a 4 g/L PM2.5 solution daily. The bacterial solution was administered at a dose of 5 × 10^6^ CFU. The HBD1 group received 25 *μ*L of a 0.04 g/L HBD1 solution daily. The inhibitor group received a dose of 10 mg/kg.

In both animal and cellular experiments, commonly used cell inhibitors include U0126, SP600125, SB203580, and BAY11-7082 (Cat. No. abs810003-25 mg, abs810008-50 mg, abs810002-10 mg, abs810013-25 mg, Absin (Shanghai) Biotechnology Co., China).

### 2.13. Data Sources

The mRNA sequencing data reported in this article were sourced from the study by Wang et al. [45]. Differentially expressed genes (DEGs) were identified based on a fold change threshold of ≥1.5 and an adjusted *p*-value of <0.05. The functional and pathway associations of these DEGs were analyzed using the Gene Ontology (GO, https://www.geneontology.org) and Kyoto Encyclopedia of Genes and Genomes (KEGG, https://www.genome.jp/kegg) databases. Data visualization was primarily conducted via the SangerBox website (https://www.sangerbox.com).

### 2.14. Statistical Methods

Data analysis was performed using GraphPad Prism 8 software (GraphPad Software Inc.), and results were expressed as mean ± standard deviation. The Student's *t*-test was employed for comparisons between two groups. For multiple data groups, one-way ANOVA followed by Newman–Keuls multiple comparisons test was utilized. Survival analysis was conducted using the log-rank test. A *p*-value of <0.05 was considered indicative of the statistical significance.

## 3. Results

### 3.1. Effects of PM2.5 on Bacterial Invasion

**Figure 2:**
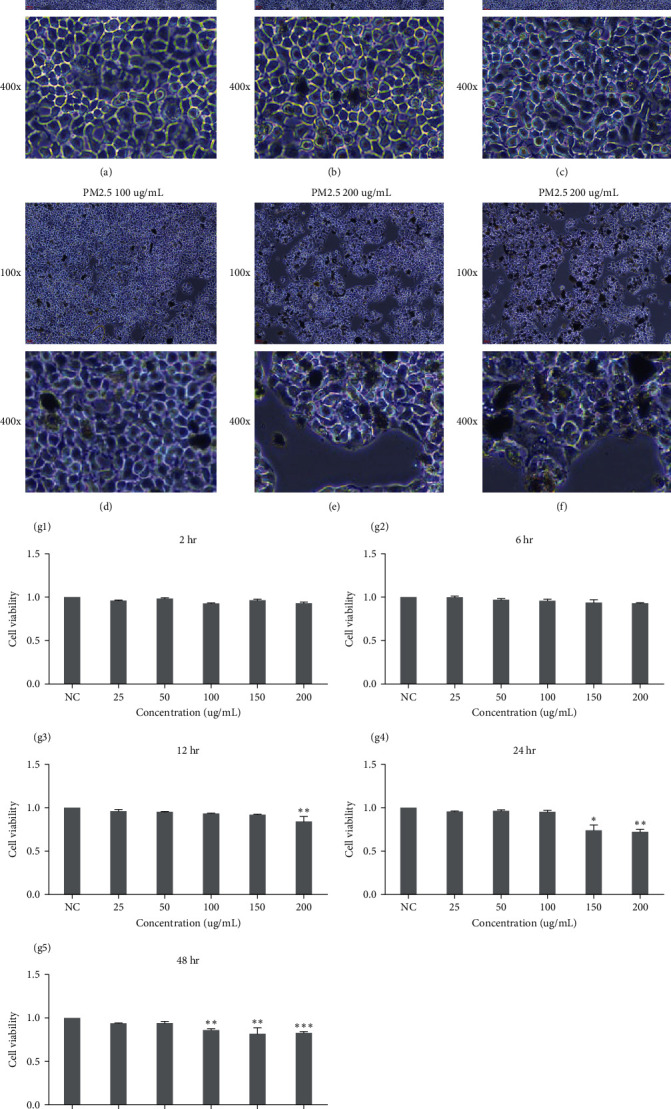
Effect of PM2.5 coculture on cell activity. (a–f) Microscopic morphological changes in HBE after exposure to different concentrations of atmospheric particulate matter PM2.5 at 24 hr. The CCK-8 method was used to detect cell viability at different concentrations of atmospheric particulate matter PM2.5 cocultured with HBE cells for different times (*n* = 3). (g) A concentration gradient of 0, 25, 50, 100, 150, 200 *μ*g/mL of PM2.5 was set within each time point. (g1–g5) represents PM2.5 treatment for 2, 6, 12, 24, and 48 hr, respectively (*n* = 3).  ^*∗*^*P*  < 0.05,  ^*∗∗*^*P*  < 0.01,  ^*∗∗∗*^*P*  < 0.001 vs. NC (*t* test) in this figure.

**Figure 3:**
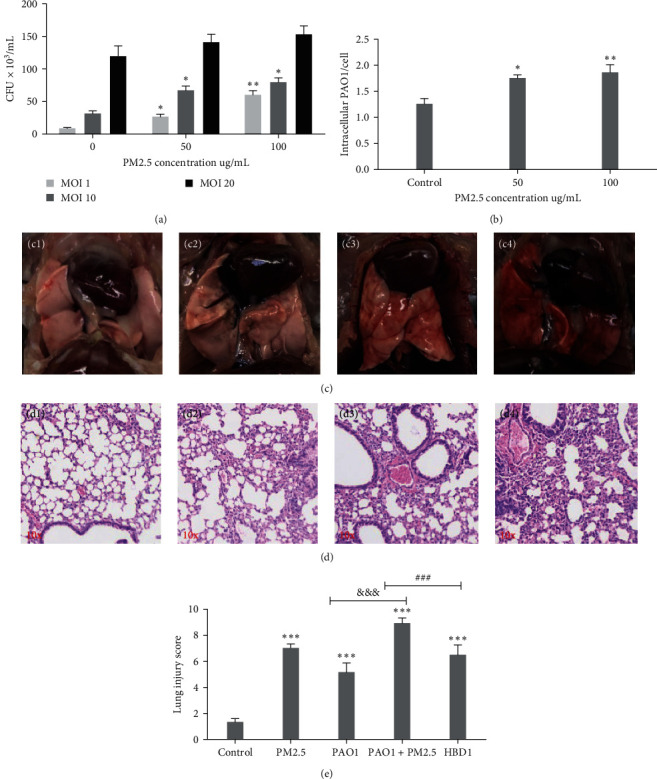
Effect of PM2.5 on bacterial infestation. (a) The effect of PM2.5 on the bacterial invasiveness of PAO1-infested HBE was examined by the colony method. Different concentrations (0, 50, 100, 200 *μ*g/mL) of PM2.5 stimulated HBE cells for 24 hr, and then the configured PAO1 bacterial solution was added to the exposure model at different infection complexes (MOI = 1, 10, 20) (*n* = 3). (b) The number of intracellular viable bacteria after PAO1 infection with HBE stimulated by different concentrations of PM2.5 (0/25/50/100 *μ*g/mL) (*n* = 3). (c) The mice were modeled with gross lung and HE staining performance (c1–c4) in the order of control, PM2.5, PAO1, and PAO1 + PM2.5 groups (*n* = 6). (d) For HE staining microscopic magnification × 40x observation (d1–d4) in order of control, PM2.5, PAO1, and PAO1 + PM2.5 groups (*n* = 6).  ^*∗*^*P*  < 0.05,  ^*∗∗*^*P*  < 0.01,  ^*∗∗∗*^*P*  < 0.001 vs. control; ^&&&^*P*  < 0.001 PAO1 + PM2.5 vs. PAO1; ^###^*P*  < 0.001 PAO1 + PM2.5 vs. HBD1 (one-way ANOVA). (e) H&E staining of mouse lung tissue for lung injury scoring (*n* = 6).

Upon 24-hr coculture with varying concentrations of PM2.5, morphological changes in cells were observed microscopically. Increased entry of PM2.5 particles into HBE cells was noted, with a rise in the number of PM2.5-engulfed cells and a decrease in overall cell count as PM2.5 concentration increased (Figure 2(a)–2(f)). After 6 hr of coculture, no significant cytotoxicity was observed in HBE cells at PM2.5 concentrations below 300 *μ*g/mL, as indicated by CCK-8 assays. However, cell viability decreased substantially after 12 hr at PM2.5 concentrations exceeding 100 *μ*g/mL (Figure 2(g)). We established PAO1-infected HBE cell models with different multiplicities of infection (MOI) and PM2.5 concentration gradients to assess the impact of PM2.5 on PAO1 invasion. A notable increase in viable bacteria from cell lysates was observed at PM2.5 concentrations of 50 *μ*g/mL or higher compared to the control group (Figure 3(a)); in the MOI 10 group, the number of viable bacteria in HBE was concentration-dependent on PM2.5. The number of colonies from HBE lysates also increased significantly at higher PM2.5 concentrations compared to the control group (Figure 3(b)). These findings suggest that PM2.5 exposure enhances *P. aeruginosa* infection.

*In vivo*, control group mice exhibited glossy white lung tissues without noticeable bleeding or granularity, while PM2.5-exposed lungs appeared dull with slight hemorrhage. Lungs from PAO1-infected and PAO1 + PM2.5 groups showed pronounced edema and hemorrhage; the PAO1 + PM2.5 group exhibited more extensive hemorrhage, reddish-black lungs, and severe pneumonia symptoms such as pleural adhesion (Figure 3(c)). These results indicate that PM2.5 also exacerbates *P. aeruginosa*-induced lung injury. Additionally, we performed H&E staining on mouse lung tissues. The PM2.5, PAO1, and PAO1 + PM2.5 groups all exhibited varying degrees of inflammatory cell infiltration, alveolar lumen exudation, slight hemorrhage, alveolar wall thickening, and vasodilation. The PAO1 + PM2.5 group was the most severe, with over 90% of the field of view showing hyperemia, bleeding, collapsed alveolar structure, and diffuse inflammatory cell infiltration. Contrastingly, control mice had more intact alveoli with minimal fluid exudation and inflammatory cell infiltration (Figure 3(d)). Pathological scoring of H&E-stained lung tissues quantified the inflammation, showing significantly higher scores in all experimental groups compared to the control group, with the PAO1 + PM2.5 group exhibiting the most remarkable increase (Figure 3(e)).

### 3.2. PM2.5 Downregulates HBD1 Expression *In Vitro*

We evaluated HBD1 secretion levels in supernatants from control, PM2.5, PAO1, and PM2.5 + PAO1 groups. The results (Figure 1(a)) revealed a significant increase in HBD1 levels in the PAO1 group compared to the control, which was inhibited by PM2.5 stimulation. HBD1 levels in the PM2.5 + PAO1 group were lower than the other groups. Additionally, *DEFB1* (Defensin Beta 1) mRNA levels in HBE cells were assessed using RT–qPCR, with results (Figure 1(b)) aligning with those from ELISA.

To determine if exogenous HBD1 could mitigate PM2.5-exposed HBE cells' predisposition to *P. aeruginosa*, we added HBD1 at a concentration of 10,000 ng/mL to the PAO1 + PM2.5 HBE cells. Intracellular viable bacteria were quantified using the plate count method. Results (Figure 1(c)) indicated a significant reduction in intracellular viable bacteria in the exogenously administered HBD1 group compared to the PAO1 + PM2.5 group.

### 3.3. PM2.5 Downregulates HBD1 Expression *In Vivo*

**Figure 4:**
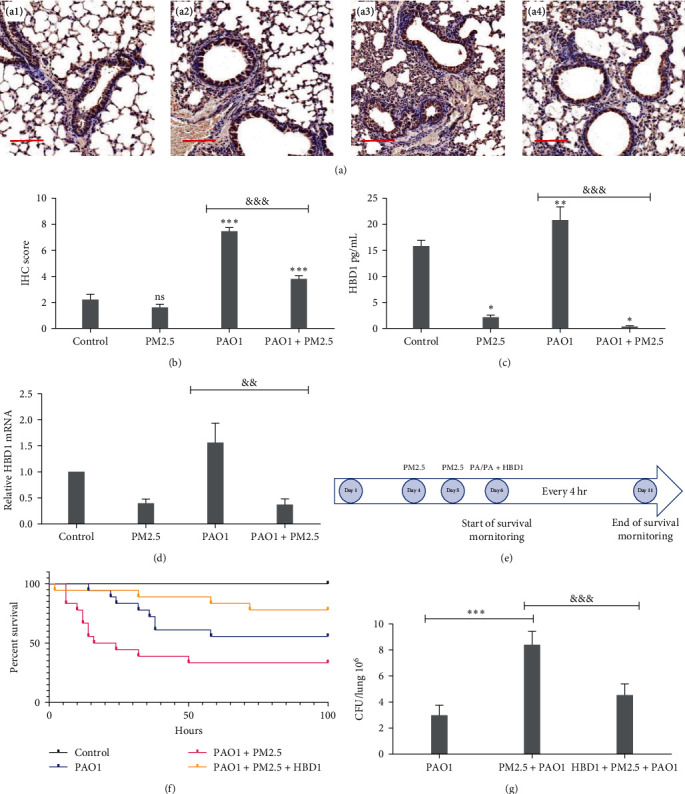
Mouse model and HBD1 expression. (a) IHC microscopic observation of mouse lung tissue HBD1, (a1–a4) in the order of control, PM2.5 group, PAO1 group, and PAO1 + PM2.5 group (scale bars = 100 *µ*m, *n* = 6). (b) IHC scoring of lung tissue in mice (*n* = 6). (c) ELISA for secreted HBD1 in the supernatant of alveolar lavage fluid (*n* = 6). (d) Detection of *DEFB1* mRNA expression in lung tissue homogenates by RT–qPCR (*n* = 6). (e) Flowchart of the animal model. (f) Survival curves for each group of mice. (g) Colony count of mouse lung grinding homogenate dilution coated plates (*n* = 6). (b–d)  ^*∗*^*P*  < 0.05,  ^*∗∗*^*P*  < 0.01,  ^*∗∗∗*^*P*  < 0.001 vs. control, ^&&^*P* <0.01, ^&&&^*P*  < 0.001 PAO1 vs. PAO1 + PM2.5 (one-way ANOVA). (g)  ^*∗∗∗*^*P*  < 0.001 PAO1 vs. PAO1 + PM2.5, ^&&&^*P*  < 0.001 PM2.5 + PAO1 vs. HBD1 + PM2.5 + PAO1 (one-way ANOVA).

Lung tissues were immunohistochemically stained (Figure 4(a)). In the control group, HBD1 was primarily expressed in airway epithelial cells and to a lesser extent in the alveolar epithelial cells. The PM2.5 group exhibited a significant decrease in HBD1 levels. The PAO1 group had the highest abundance of HBD1-expressing cells, with extensive HBD1 presence. The PAO1 + PM2.5 group had more HBD1 expression in airway and alveolar epithelium than the control group. Quantitative IHC scoring showed higher scores in the experimental group than in the control group, with the highest score in the PAO1 group (Figure 4(b)). This suggests that PAO1 stimulates HBD1 levels in lung epithelial tissues in mice, but PM2.5 exposure reduces these elevated levels.

BALF was collected from mice, and the level of secretory HBD1 in the lungs was assessed using ELISA. The results (Figure 4(c)) showed significantly lower secretory HBD1 in the PM2.5 and PAO1 + PM2.5 groups than in the control and PAO1 groups. Additionally, total mRNA from mouse lung tissues was extracted using TRIzol reagent and analyzed via RT–qPCR. The results (Figure 4(d)) were consistent with those from ELISA.

The modeling flowchart is presented (Figure 4(e)). The survival curve showed a statistically significant decrease in the PAO1 and PAO1 + PM2.5 groups compared to the control group (Figure 4(f), Table 1), and a significant improvement in the PAO1 + PM2.5 + HBD1 group compared to the PAO1 + PM2.5 group. The survival rates primarily declined in the first 50 hr of observation, with the PAO1 group's survival rates around 50%–60%, while the PAO1 + PM2.5 group's rates dropped below 50%, showing a statistically significant difference. Exogenous HBD1 administration notably improved survival rates from below 50% to around 75% and significantly reduced pathological scores in the PAO1 + PM2.5 group (Figure 3(e)).

To assess viable bacteria in murine lung tissues, lung tissues were homogenized and plated. Results (Figure 4(g)) demonstrated that PM2.5 exposure increased the number of viable PAO1 bacteria in murine lungs, while airway HBD1 administration inhibited bacterial proliferation.

### 3.4. Effect of PM2.5 on MAPK and NF-*κ*B Pathways

**Figure 5:**
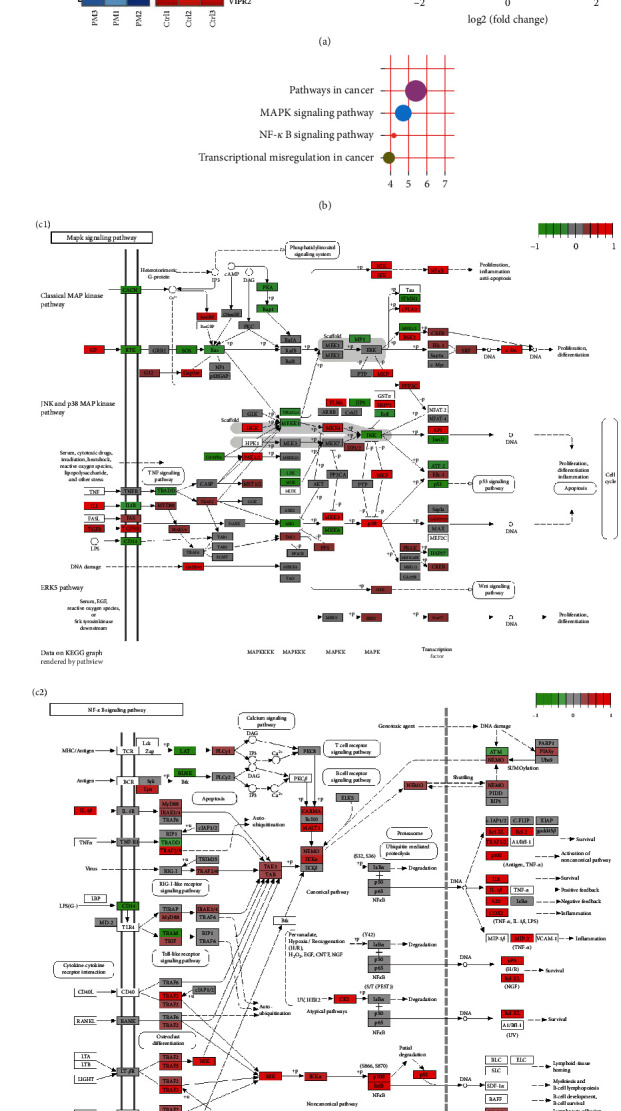
mRNA-seq results of KEGG pathway enrichment analysis and western blot results. (a) mRNA sequencing results of cells exposed to PM2.5, (a1) heat map and (a2) volcano map (*n* = 3). (b) KEGG pathway enrichment analysis. Upregulated genes are mainly tumor, MAPK, and NF-*κ*B pathway-related genes (*n* = 3). (c) Signaling pathway related genes change: (c1) MAPK pathway and (c2) NF-*κ*B pathway (*n* = 3). (d) Western blot detected the expression of MAPK pathway and NF-*κ*B pathway proteins, and the experimental groups were control group, PM2.5 group, PAO1 group, and PAO1 + PM2.5 group. Among them, i*κ*B and P65 were NF-*κ*B pathway proteins, P44, P38, and JNK were MAPK pathway proteins, and *β*-actin and lamin-B were internal reference proteins (*n* = 3).

mRNA high-throughput sequencing of PM2.5-exposed HBE cells was performed. Results (Figure 5(a)) showed that 666 genes were upregulated while 817 were downregulated. KEGG metabolic pathway enrichment analysis (Figures 5(b) and 5(c)) revealed that upregulated genes were closely linked to cancer, MAPK, and NF-*κ*B pathways. This suggests that genes related to the MAPK pathway and most NF-*κ*B pathway-related genes were activated, likely contributing to the downregulation of HBD1.

Proteins from PM2.5-exposed HBE cells were extracted and analyzed for MAPK and NF-*κ*B pathway-related protein expression using western blot. Results (Figure 5(d)) indicated that the NF-*κ*B pathway-related protein, i*κ*B, was downregulated in the PAO1 and PAO1 + PM2.5 groups, while no significant change was observed in P65 expression. MAPK pathway-related proteins were upregulated in the PM2.5, PAO1, and PAO1 + PM2.5 groups compared to the control group, with more pronounced upregulation in the PAO1 and PAO1 + PM2.5 groups than in the PM2.5 group. The PAO1 + PM2.5 group showed less upregulation of P44 and JNK compared to the PAO1 group. These results demonstrate that PM2.5 activated the MAPK and NF-*κ*B pathways.

### 3.5. PM2.5 Downregulates HBD1 Expression through MAPK/NF-*κ*B Pathway *In Vitro*

**Figure 6:**
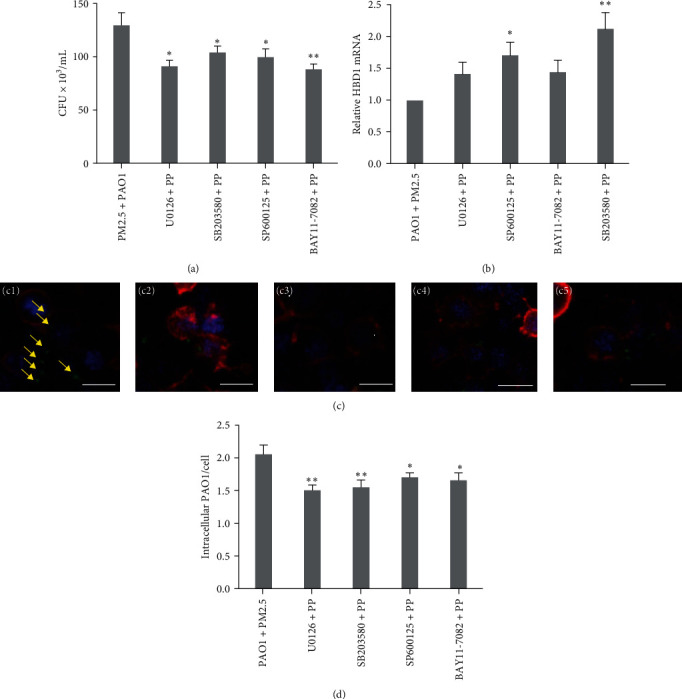
Changes in bacterial invasiveness in the PM2.5+PAO1 cell model in the presence of pathway inhibitors. (a) Cell lysate dilution coating plate colony count (*n* = 3). (b) Detection of *DEFB1* mRNA expression in lung tissue homogenates by RT–qPCR (*n* = 3). (c) Laser confocal microscopy photographed intracellular live bacterial infection in each group under the effect of inhibitors. Yellow arrows indicate PAO1. (c1–c5) PAO1 + PM2.5 group; U0126 + PAO1 + PM2.5 group; SB203580 + PAO1 + PM2.5 group; SP600125 + PAO1 + PM2.5 group; BAY11-7082 + PAO1 + PM2.5 group, in that order (scale bars = 20 *µ*m, *n* = 3). (d) The number of viable intracellular bacteria was counted under laser confocal microscopy (*n* = 3).  ^*∗*^*P*  < 0.05,  ^*∗∗*^*P* < 0.01,  ^*∗∗∗*^*P* < 0.001 vs. PM2.5 + PAO1 (*t* test).

Administration of MAPK pathway inhibitors (SB203580/SP600125) and the NF-*κ*B pathway inhibitor BAY11-7082 significantly reduced CFU in plate colony counts from cell lysates (Figure 6(a)).

RT–qPCR results (Figure 6(b)) suggested that *DEFB1* mRNA expression was significantly elevated in MAPK pathway-inhibited groups (SP600125 and SB203580). In contrast, treatment with the MAPK pathway inhibitor U0126 and NF-*κ*B pathway inhibitor BAY11-7082 did not result in elevated *DEFB1* mRNA levels.

Laser confocal microscopy revealed a remarkable decrease in intracellular viable bacteria in MAPK pathway-inhibited groups (U0126/SB203080/SP600125) and NF-*κ*B pathway-inhibited groups (BAY11-7082) compared with the control group. The results (Figures 6(c) and 6(d)) showed a reduction in intracellular PAO1 in all groups. Therefore, we concluded that PM2.5 downregulates *DEFB1* expression through the MAPK/NF-*κ*B pathway *in vitro*.

### 3.6. PM2.5 Downregulates HBD1 Expression through MAPK/NF-*κ*B Pathway I*n Vivo*

**Figure 7:**
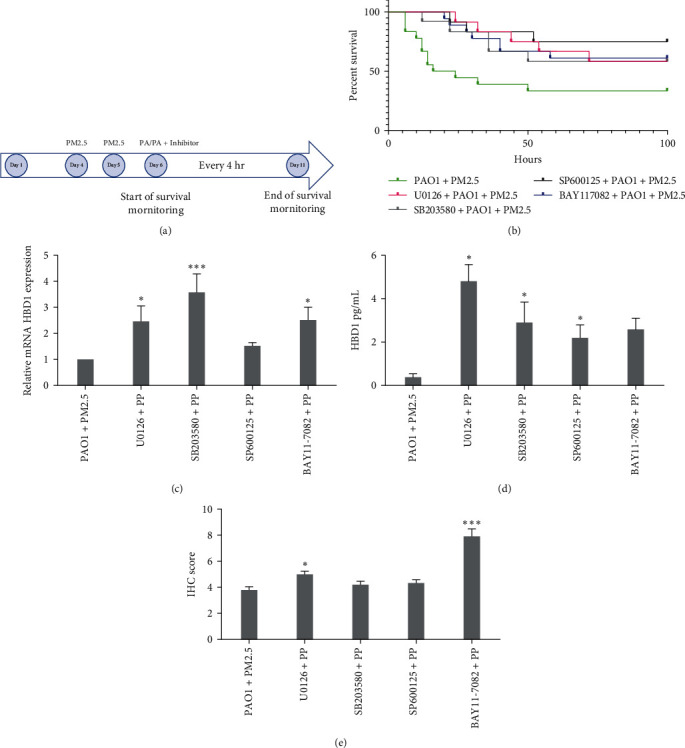
Mouse model and HBD1 expression under inhibitor action. (a) Flowchart of the animal model. (b) Survival curves in each group under the effect of inhibitors. (c) Detection of *DEFB1* mRNA expression in lung tissue homogenates by RT–qPCR (*n* = 6). (d) ELISA for secreted HBD1 in the supernatant of alveolar lavage fluid (*n* = 6). (e) IHC scoring of lung tissue in mice (*n* = 6).  ^*∗*^*P*  < 0.05,  ^*∗∗*^*P*  < 0.01,  ^*∗∗∗*^*P*  < 0.001 vs. PM2.5 + PAO1 (*t* test).

The modeling flowchart is depicted in Figure 7(a). Analysis of the survival curve indicated a statistically significant enhancement in survival rates for the groups treated with SP600125 and BAY11-7082 compared to the control group (Figure 7(b), Table 2). Furthermore, the PAO1 + PM2.5 + HBD1 group exhibited a marked improvement in survival compared to the PAO1 + PM2.5 group.

RT–qPCR analysis of lung tissue from mice treated with inhibitors showed an increase in *DEFB1* mRNA expression across all MAPK/NF-*κ*B-inhibited groups. Notably, this upregulation was statistically significant in the U0126, SB203580, and BAY11-7082 groups relative to the control group (PM2.5 + PAO1; Figure 7(c)).

The HBD1 concentration in BALF was significantly higher in the MAPK inhibitor groups (U0126/SB203580/SP600125) compared to the control group (PM2.5 + PAO1) as illustrated in Figure 7(d). However, the increase in HBD1 secretion was not significant in the NF-*κ*B inhibitor-treated group.

IHC staining of lung tissues from mice treated with the inhibitors demonstrated that IRC were elevated in all inhibitor groups compared to the control group (PM2.5 + PAO1). The increase was statistically significant in the U0126, SB203580, and BAY11-7082 groups, as shown in Figure 7(e).

## 4. Discussion

Air pollution, with PM2.5 as a key component, has been acknowledged globally as a critical environmental issue [46]. PM2.5 exposure is implicated in the etiology of numerous diseases [47–49].

High concentrations of PM2.5 in the environment can disrupt the balance of the human respiratory tract microbiota, altering the richness, evenness, and composition of the microbiome in mice's respiratory tracts [23, 50] and increasing susceptibility to pathogenic microorganisms like pneumococcus [51]. High PM2.5 levels also directly enhance the growth rate of opportunistic pathogens such as *Escherichia coli* and *P. aeruginosa*, significantly increasing the production of their biofilms and altering the virulence of these pathogens [52]. Our study results show that in the PM2.5-exposed *in vitro* model, the number of live *P. aeruginosa* per airway epithelial cell is higher compared to the unexposed group. *In vivo* models reveal more severe lung damage in PM2.5-exposed mice than in unexposed ones, with greater lung injury and higher numbers of viable bacteria per unit lung tissue, and lower survival rates after *P. aeruginosa* infection. This is consistent with the previous findings, revealing that PM2.5 exposure increases the invasiveness of *P. aeruginosa* on airway epithelia, exacerbates lung damage caused by infection, and reduces survival time after infection.

There are many reported mechanisms by which PM2.5 increases bacterial invasion, such as impairing macrophage function, reducing lung natural killer cells, and activating chronic inflammation in the lungs [24–31, 53]. However, the impact of PM2.5 exposure on the expression of defensins is inconsistent [54, 55]. Our study found that after HBE cells are exposed to PM2.5, HBD1 is reduced at all expression levels. PM2.5-exposed airway epithelial cells show a higher number of invasive live bacteria intracellularly compared to the unexposed infection group. Administering exogenous HBD1 improves the situation of bacterial invasion in the *in vitro* model post-PM2.5 exposure, and in the mouse model, not only is the invasion improved, but lung injury is reduced, and survival time postinfection increases. HBD1 is a key component of innate immunity, regulating the composition of the body's microbiota without inducing antibiotic resistance like antibiotics do [56]. Although HBD1 is constitutively expressed in tissues, its expression level is regulated by various factors. In colorectal cancer models, HBD1 expression is suppressed [57] and can be enhanced by administering immune boosters [58]. HBD1 levels are also affected by the body's infection status, with high expression in urethral and gingival epithelial cells during the early innate immune response to viral and bacterial infections [59, 60]. Our study reveals that PM2.5 exposure downregulates HBD1 expression, thereby increasing the invasiveness of *P. aeruginosa*. Furthermore, after PM2.5 exposure followed by *P. aeruginosa* contact, HBD1 expression cannot return to levels seen in simple *P. aeruginosa* infections. This implies that under real environmental conditions, with PM2.5 exposure, the immune processes represented by HBD1 are suppressed, making it more difficult to control bacterial invasion in the respiratory tract.

Studies have shown that *in vitro* exposure to soluble PM2.5 extracts can reduce the vitality of airway epithelial cells and increase apoptosis, with soluble PM2.5 extracts inducing oxidative stress and enhancing pro-inflammatory factor expression by activating the NF-*κ*B and MAPK signaling pathways [61]. PM2.5 can also activate the ATR-CHEK1/CHK1 pathway in airway epithelia, leading to TP53-dependent autophagy and VEGFA production, activating chronic inflammation in the airway epithelia [62]. Our study indicates that in the PM2.5-exposed *in vivo* model, MAPK pathway proteins P44 and JNK are upregulated, while the NF-*κ*B pathway inhibitory protein i*κ*B is downregulated. Adding MAPK and NF-*κ*B pathway inhibitors reduces the number of invasive live bacteria in single cells in the *in vitro* model post-PM2.5 exposure compared to groups without pathway inhibitors, with improved invasion situations, reduced lung injury, and increased survival time postinfection in *in vitro* experiments with inhibitors. Qian et al.'s [63] study demonstrated that cigarette smoke can downregulate HBD1 levels in oral mucosal epithelial cells by activating the NF-*κ*B pathway, consistent with our findings. Thus, we reveal for the first time that PM2.5 exposure downregulates HBD1 in the respiratory tract, predisposing cells to bacterial invasion via MAPK and NF-*κ*B pathway activation.

Our study, however, has limitations: (1) It focuses primarily on HBD1 levels in HBE cells, with no significant PM2.5-induced changes observed in alveolar epithelial cells (A549), and the effects on other cell types remain unexplored. (2) We used *P. aeruginosa*, a common bacterium in hospital-acquired pneumonia (HAP), but the effects of PM2.5 on other pathogens, like *Streptococcus pneumoniae* prevalent in community-acquired pneumonia (CAP), warrant further investigation. (3) Our research lacks direct clinical evidence to support our findings.

Despite these limitations, this study illuminates the pathogenesis and potential clinical interventions for respiratory infections related to PM2.5 exposure. It provides a theoretical foundation for using HBD1 as a potential antimicrobial agent against PM2.5-induced infections, facilitating the development of therapeutic strategies targeting MAPK and NF-*κ*B pathways, and offering experimental insights into the mechanisms for mitigating airway inflammation and restoring mucosal immune functions.

## 5. Conclusions

In conclusion, this study confirmed the toxic effects of PM2.5 and established that PM2.5 enhances *P. aeruginosa* bacterial invasion by diminishing HBD1 levels, a process that can be counteracted with exogenous HBD1. Furthermore, our findings revealed that PM2.5 suppresses *DEFB1* expression through the activation of MAPK and NF-*κ*B pathways. Additionally, inhibiting these pathways was shown to elevate HBD1 levels and reduce bacterial invasion.

## Figures and Tables

**Table 1:** *P*-value after log-rank statistics for survival data for each group of mice (*n* = 10, log-rank test).

Control group	Treatment group	Log-rank *P*-value
Blank	PAO1	0.005
Blank	PAO1 + PM2.5	<0.001
Blank	PAO1 + PM2.5 + HBD1	0.065
PAO1 + PM2.5	PAO1 + PM2.5 + HBD1	0.004

**Table 2:** *P*-value after log-rank statistics for survival data for each group of mice (*n* = 10, log-rank test).

Control group	Treatment group	Log-rank *P*-value
PAO1 + PM2.5	U0126 + PAO1 + PM2.5	0.062
PAO1 + PM2.5	SP600125 + PAO1 + PM2.5	0.016
PAO1 + PM2.5	SB203580 + PAO1 + PM2.5	0.090
PAO1 + PM2.5	BAY117082 + PAO1 + PM2.5	0.029

## Data Availability

The datasets used and/or analyzed during the current study are available from the corresponding author on reasonable request.
